# The Psychological Impact of Coronavirus Pandemic Restrictions in Italy. The Mediating Role of the Fear of COVID-19 in the Relationship between Positive and Negative Affect with Positive and Negative Outcomes

**DOI:** 10.3390/ejihpe11030050

**Published:** 2021-07-08

**Authors:** Andrea Zammitti, Chiara Imbrogliera, Angela Russo, Rita Zarbo, Paola Magnano

**Affiliations:** 1 Department of Educational Sciences, University of Catania, 95131 Catania, Italy; 2 Faculty of Human and Social Sciences, Kore University, 94100 Enna, Italy; chiara.imbrogliera@unikorestudent.it (C.I.); angela.russo13@unikorestudent.it (A.R.); rita.zarbo@unikore.it (R.Z.); paola.magnano@unikore.it (P.M.)

**Keywords:** COVID-19, positive affect, negative affect, spiritual well-being, flourishing, perception of PTSD symptoms

## Abstract

Italy was quickly hit hard by the coronavirus. ‘Lockdown’ has significantly impacted the psychological health, personal wellbeing and quality of life of the people. The study aims to explore the relationship between positive and negative affect, as well as positive (spiritual well-being and flourishing) and negative outcomes (psychological distress caused by a traumatic life event in terms of perception of PTSD symptoms) on Italian adults during the lockdown period. Data was collected between April and May 2020. The participants were 281 Italian adults aged between 18 and 73 years. The survey was composed of the following measures: Flourishing Scale, Jarel Spiritual Well-Being scale, Positive and Negative Affect Schedule, Impact of Event Scale—Revised, Fear of COVID-19. The mediational analysis shows that fear of COVID-19 fully mediates the relationship between negative affect and spiritual well-being and flourishing; fear of COVID-19 partially mediates the relationship between negative affect and PTSD symptoms; the positive affect shows only direct effects on positive outcomes. Therefore, fear of COVID-19 does not play any mediation role. Implications for psychological interventions and future research will be discussed.

## 1. Introduction

After the first cases of Severe Acute Respiratory Syndrome in Wuhan city, the World Health Organization [1] ascertained the existence of a disease, naming it COVID-19 [1]. This disease has spread rapidly around the world and has become a global public health threat [2]. Many countries have adopted prevention measures: physical distancing practices, distance working, lockdown, self-isolation or cancellation of flights [3]. As a result, the global economy has suffered a severe setback; in fact, the International Monetary Fund (IMF) has called all this “the Great Lockdown” [4], and its impact on the world will be «the worst economic downturn since the Great Depression» [5].

The pandemic situation has also affected people’s lives. Individuals experienced feelings of fear, and anxiety during the pandemic [6]. Fear is an emotion caused by the perception of a threat and allows animals to deal with dangers; therefore, it performs an adaptive function [7]. In humans, fear can reach high levels and arouse further negative emotions [8,9], increasing the risk of physical and psychopathological problems [10]. Pandemics cause fear responses as they are invisible viruses that are transmitted between people, increasing the risk of mortality [11,12]. Fear causes an inability to act, stress and negative feelings [13]. People suffering from anxiety during a pandemic have high levels of post-traumatic stress, general stress, anxiety, health anxiety, and suicidal thoughts [14,15,16,17]. Some authors have argued that the importance of the dimensions of positive psychology is evident during the epidemic period [18].

This suggests that psychological support interventions are needed [19]. Some authors have stressed the importance of providing psychological support to healthcare professionals, improving their ability to address the anxiety associated with the disease [20]. However, due to the worries and anxiety that affect all people, other authors have pointed out the need for governments to offer psychological support to citizens during a pandemic, for example, by providing home interventions [21], including people presenting with a vulnerability [22].

In the Italian context, a recent study conducted on the adult population showed that only 5% of the respondents considered the possibility that they would contract a disease caused by a novel virus; this means that, in addition to psychological interventions, it was necessary to empower the population regarding prevention and containment measures [23]. In the initial phase of the pandemic, Italians who were emotionally stable were more resistant to concerns about COVID-19 [24]. However, other studies have shown that 5.1% of 1,639 respondents reported PTSD symptomatology [25]. In the same contest, Ceccato and colleagues [26] evaluated the role of the individual’s Balanced Time Perspective (BTP) on expectations for the future, showing that people with higher levels of BTP had fewer negative beliefs about COVID-19’s future. The quarantine experience during the pandemic had a significant psychological impact at all ages, starting from adolescence [27]. This study aims to contribute to the research on the psychological effects of the COVID-19 pandemic on the Italian population, focusing on both positive and negative psychological dimensions.

### 1.1. Positive and Negative Affects

Affect is a broad concept that covers feelings that are accessible to consciousness and are present in many affective events including emotions, physical sensations, attitudes, moods, and affective traits [28]. Negative and positive affects are considered the two predominant dimensions of mood [29,30]. Several researchers have attempted, over the years, to understand the nature of the relationship between positive and negative affects. The more ancient literature on negative and positive affect [31,32,33] has focused on the possibility that they are bipolar dimensions (opposite poles of the same continuum). The more recent studies, on the contrary, have shown the non-correlation of the two dimensions, suggesting that they are independent constructs [34,35,36]. Positive and negative affect refer, respectively, to positive emotional states such as feeling joy, interest, confidence or alertness and negative emotional states such as feeling fear, sadness, anger, guilt, contempt or disgust [37].

In accordance with what is advocated by positive psychology [38], Kuppens, Realo, and Diener [39], relying on data from a cross-cultural study involving as many as 46 different nations around the world, showed that positive affective experiences are strongly linked to life satisfaction. In addition, many studies confirm that the ratio of positive to negative affect has important implications for subjective well-being and flourishing [40,41,42,43]. The presence of positive affect also plays a key role as a coping mechanism, as it helps to deal with stressful situations [44] and can lead to building lasting resilience to better cope with future stressful events [45,46,47]. Moreover, results from positive affect enhancement interventions showed that changes in affect were also associated with changes in self-efficacy, job satisfaction, relationship satisfaction, and mental health [48]. On the contrary, a prevalence of negative affect, even in the absence of objectively stressful circumstances, is associated with low self-esteem, dissatisfaction, stress, and physical symptoms [49].

In sum, affects can interact with important areas of human life, but they can also be influenced by circumstances or contextual factors such as positive or negative daily events (e.g., the occurrence of positive daily events, even in the presence of negative daily events can mitigate the negative affects) [50]. Just like the onset of the COVID-19 pandemic, the effects of situational stress are excellent examples of unexpected events that involve the activation of positive or negative affect and emotional experiences [51].

### 1.2. Relationships between Psychological Distress and COVID-19

Psychological distress is a broader manifestation of mental-health-related problems, which is characterized by symptoms of depression, anxiety, and stress-related concerns; it is known that psychological distress shows an increasing trend in terms of severity [52]. In a systematic review and meta-analysis, including 68 studies comprising 288,830 participants from 19 countries, anxiety symptoms were the most prevalent, especially among people with lower socioeconomic status and among women [53]. A study conducted in Spain during the COVID-19 pandemic, on a sample of 3055 participants [54], showed that 36.6% of them presented psychological distress in terms of symptomatic responses related to the perception of symptoms of Post-Traumatic Stress Disorder (avoidance, intrusion, and hyperarousal). Indeed, negative psychological outcomes are common during a crisis [55], including the perception of PTSD symptoms. For example, during pandemics, people tend to develop numerous types of worry, and avoidance coping responses [56]. Avoidance, together with thoughts or intrusive imagery, constitutes one of the variables that form the COVID stress syndrome [57,58]. The COVID-19 pandemic has also elicited psychological reflexes of hyper-arousal [59]. Together, these contribute to increasing levels of uncertainty, fear, anxiety and stress [60]; experience related to previous pandemics confirms that health problems and post-traumatic stress diagnoses are increasing [15]. Sun et al. [61] collected survey data from a general sample of adults in mainland China and found that 4.6% of the people (who were part of the study) displayed symptoms suggesting a probable PTSD diagnosis. However, the number of probable PTSD diagnoses was significantly higher (18.4%) in a subsample of participants with greater exposure to COVID-19 (i.e., participants with suspected or confirmed COVID-19 diagnoses and those who have had close contact with a person who had COVID-19). The authors reported that poor sleep quality, being a woman, and recent exposure to Wuhan city (e.g., living in or traveling to Wuhan) were connected with increased PTSD symptomatology.

People with this symptomatology are also forced to relive the negative effects caused by a traumatic event, which can cause subsequent avoidance behaviors toward trauma-related stimuli; these symptoms have an important impact on people’s daily life and work [62,63]. With the extremely high infection concerns, enough evidence has demonstrated that COVID-19 was considered as a life-threatening public health emergency, and a disease serious enough to cause PTSD. 

### 1.3. Relationship between Flourishing and Affects

The different literature reviews [64,65,66] have explored the relationship between emotions and wellbeing, showing that positive emotions, often mild and ephemeral, both reflect and produce wellbeing [67]. In the literature, human well-being has been conceptualized both through the hedonic approach and the eudaemonic approach [64,68], although recently the two constructs have been integrated due to their overlap [69]. On the one hand, eudemonic wellbeing coincides with flourishing [70], a recent and developing construct [71], which refers to social–psychological prosperity, exemplified by the optimal human functioning, such as positive relationships, feelings of competence and having meaning and purpose in life [68,72,73]. On the other hand, hedonic or subjective well-being concerns the presence of positive emotions such as happiness and wish satisfaction [44,74], and is strongly influenced by the overall balance between people’s positive and negative emotions. Positive emotions can definitely be considered both as markers of flourishing [27] and enhancers of flourishing [75]. 

Therefore, it might be interesting to better understand what can influence the relationship between emotions and flourishing during the COVID-19 quarantine experience.

### 1.4. Relationship between Spiritual Wellbeing and Affects

Spiritual wellbeing is a multidimensional construct that incorporates both existential and religious dimensions [76,77] and could be defined as a sense of harmonious interconnectedness between the self, others and nature, which exists throughout and beyond time and space, and leads to a realization of the ultimate purpose and meaning of life [78].

It is well known that life stress predicts negative affect [79,80] and that personal spirituality could be a useful resource for maintaining life satisfaction in the face of stressors [81]; nevertheless, previous research has shown that having a more spiritually integrated life does not significantly relate to one’s affective experience [81,82].

Despite this, spiritual wellbeing was demonstrated to be positively correlated with positive affective states and adequate stress-coping strategies [83,84]. Furthermore, many authors [85] stated that religion and spirituality can play an important role in the relief of suffering, with an influence on health outcomes and a minimization of the consequences of social isolation during the COVID-19 pandemic. In an uncertain future, with little control over what happens next, the only certainty for many people lies in their religious faith, which is an essential element that people need to stay healthy [86]. Deepening the relationship between positive emotions and spiritual wellbeing, Van Cappellen et al. [87] showed that positive emotions and, more specifically, self-transcendent positive emotions (such as awe, gratitude, love, and peace) play a mediation role between spirituality and wellbeing. It seems that putting faith into action can help people maintain health and wellbeing [88].

Therefore, it might be interesting to better understand what can influence the relationship between emotions and spirituality during the COVID-19 pandemic.

## 2. Aims of Research

Starting from an analysis of the existent literature, given that positive and negative affect are related to positive and negative individual outcomes, we aim to explore the effects that positive and negative affect have on positive outcomes, such as spiritual well-being and flourishing, and negative outcomes, such as psychological stress, expressed in terms of self-assessment of the symptoms of post-traumatic stress disorder (PTSD), verifying if these relationships are mediated by fear of COVID-19. In more detail, we hypothesize the following:

**Hypothesis** **1a** **(H1a).** *Positive and negative affect directly predicts PTSD;*

**Hypothesis** **1b** **(H1b).** *Positive and negative affect predicts PTSD through mediation of the fear of COVID-19;*

**Hypothesis** **2a** **(H2a).** *Positive and negative affect directly predicts spiritual well-being;*

**Hypothesis** **2b** **(H2b).** *Positive and negative affect predicts spiritual well-being through mediation of the fear of COVID-19;*

**Hypothesis** **3a** **(H3a).** *Positive and negative affect directly predicts flourishing;*

**Hypothesis** **3b** **(H3b).** *Positive and negative affect predicts flourishing through mediation of the fear of COVID-19.*

## 3. Materials and Methods

### 3.1. Research Design

We conducted a cross-sectional study, measuring all the variables at the same time, through administration of an online survey. The online survey was administered individually and anonymously in the period between March and June 2020, during the first lockdown due to the COVID-19 pandemic; the participants came from different Italian regions. The participants were selected from the general population on the basis of proximity to the researchers and their collaborators. In addition, psychological disorders were used as an exclusion criterion; the participants were asked if they had received a diagnosis of a psychological disorder in the past six months.

The survey was advertised as a research study, designed to investigate some thoughts, emotions and behaviors that people experienced in that moment of health emergency. The respondents gave their consent to participate before starting and were free to interrupt their participation at any point. 

The respondents participated on a voluntary basis, and they could deny or withdraw participation at any point during the research. The respondents provided an individual code composed of the first three letters of their first name and the first three letters of their last name, which, matched with other demographic information, allowed us to check the eventual double compilation. The survey was approved by the ethical commission of the universities involved, and followed the ethical rules of the Italian Psychological Association.

### 3.2. Measures

Positive and Negative Affect Schedule (PANAS) [89,90]. 

PANAS comprises 20 items, 10 of which measure the “positive affect”, and 10 the “negative affect”. The scale consists of a number of words that describe different feelings and emotions (e.g., “interested”, “scared”, “afraid”). Participants were asked to indicate how they felt in recent weeks. The answers were given using a 5-point Likert scale to indicate the “intensity”, which varied from 1 (not at all) to 5 (a lot). In the study conducted by Watson, Clark and Tellegen [81], Cronbach’s alpha was 0.86 for Positive Affect and 0.87 for negative Affect. The correlation between the scales was −0.09. In the Italian version of PANAS [90], Cronbach’s alpha coefficients correspond to the original scales. The Cronbach’s alpha for the study sample was 0.85 for positive affect, and 0.87 for negative affect.

Fear of COVID-19. 

We used three items, previously used in other research, to assess fear of SARS or AIDS [16,91]. Participants were asked to read the statements and give an answer on a 5-point Likert scale, from 1 (not at all) to 5 (very). The three items were: “Thinking about COVID-19 makes me feel anxious”, “I feel tense when I think about the threat of COVID-19” and “I feel quite anxious about the possibility of another outbreak of COVID-19”. In Wu et al.’s [16] study, the Cronbach’s alpha of the scale was 0.70. Cronbach’s alpha for the study sample was 0.93.

Impact of Event Scale—Revised (IES-R) [92,93]. 

This scale is probably the most-used self-report measure of traumatic stress [94]. It is not a diagnostic scale for PTSD, but is used to evaluate the response after a traumatic event [95]. Aljaberi and colleagues [96] showed that IES-R is a reliable instrument for evaluating traumatic distress related to the COVID-19 pandemic. We used the scale to assess perceived stress in relation to the COVID-19 pandemic. Participants were asked to read statements describing stressful situations in reference to the COVID-19 pandemic. A sample item is as follows: “Any reminder brought back feelings about it”. Participants were asked to indicate how well the statements fit with what they felt during the last week, on a 5-point Likert Scale ranging from 1 (not at all) to 5 (a lot). In the Italian version of the IES-R [93], the measure showed satisfactory values of internal consistency, with a Cronbach’s alpha value of 0.83 for the total score. Cronbach’s alpha for the study sample was 0.90.

Jarel Spiritual Well-Being Scale [72,97]. 

The original version is composed of 21 items; the Italian adaptation uses 16 items with a 5-point Likert Scale ranging from 1 (strongly disagree) to 5 (strongly agree). A sample item is as follows: “I find meaning and purpose in my life”. In the original version [89], the authors identified three factors: Faith and Belief, Life and Self-Responsibility, and Life Satisfaction and Actualization. Cronbach’s alphas were, respectively, 0.77, 0.54, and 0.76. In the Italian study [72], the authors identified the following factors: Faith and Belief (Cronbach’s alpha was 0.88), Meaning of Life (Cronbach’s alpha was 0.80), and Quality of Relationships (Cronbach’s alpha was 0.64). Cronbach’s alpha for the total point of Spiritual Well-Being was 0. 81. For the study sample, the Cronbach’s alpha of the total score was 0.85.

Flourishing Scale [68]. 

This is a one-dimensional instrument designed to measure flourishing, consisting of eight items. Participants were asked to read the statement and their answers were given using a 7-point Likert scale ranging from 1 (strongly disagree) to 7 (strongly agree). Sample item is “I am a good person and live a good life”. In Diener et al.’s [68] study, the Cronbach’s alpha of the scale was 0.87. The Cronbach’s alpha for the study sample was 0.88.

### 3.3. Participants

The participants were 281 Italian adults (male = 90, 32%; female = 191, 68%) aged between 18 and 73 years (M = 34.09; SD = 13.02). According to our sample size calculation, our sample is capable of reflecting the target population with a confidence level of 95% at a 5.81% confidence interval. The participants were recruited from the general population, through convenience sampling. The majority were workers (153, 54.45%); the remaining portion was composed of students (98, 36.30%) or unemployed people (26, 9.25%). 

### 3.4. Data Analysis

To conduct data analysis, we used a path analysis. We verified the mediation hypothesis through Jamovi 1.6.23, which shows the significance of the indirect effects using the bootstrapping method, with 5000 repetitions. The mediation outcomes are reported, presenting the completely standardized β, and the confidence intervals (C.I.) 95%, which indicate the significance of the effect with a 5% of probability of error (a C.I. that does not comprise 0 is significant).

Finally, other well-known statistical analyses were conducted using SPSS 25.0. Missing values for the relevant items were estimated using the expectation maximization method. None of the items had more than 5% missing values, indicating the appropriateness of this option.

## 4. Results

### 4.1. Descriptive Statistics and Correlations

Table 1 shows the mean and standard deviation of the measures (positive affect, negative affect, the fear of COVID-19, PTSD symptoms, spiritual wellbeing, and flourishing) and the correlations between the variables calculated through Pearson’s *r*. Positive affect is positively and strongly correlated with spirituality and flourishing, and negatively and moderately correlated with PTSD symptoms and the fear of COVID-19; conversely, negative affect is negatively, but moderately, related to spirituality and flourishing and positively and strongly related to PTSD symptoms and the fear of COVID-19. Moreover, the fear of COVID-19 is positively and strongly associated with PTSD symptoms.

### 4.2. Mediational Analysis

**Figure 1:**
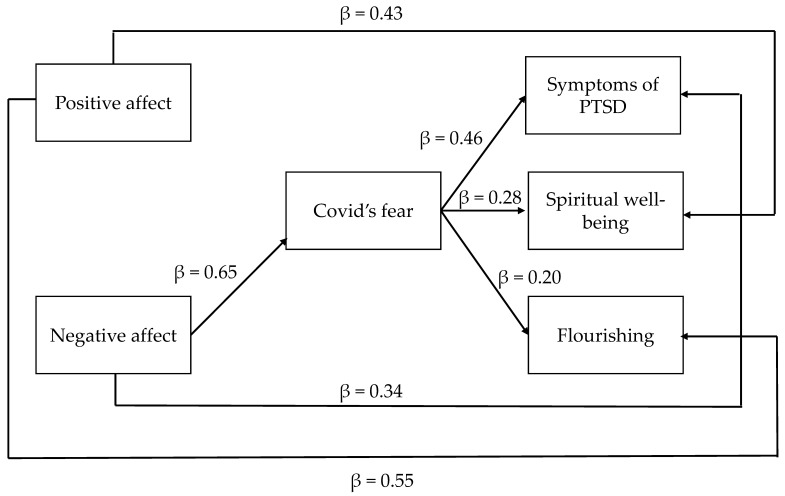
Path model for positive and negative affects.

We tested our hypotheses using a path analysis. The model had positive and negative affect as antecedents, the mediator was fear of COVID-19 and the outcomes were PTSD symptoms as a negative outcome, and spiritual wellbeing and flourishing as positive outcomes. The final model is presented in Figure 1. In the model, having contracted COVID-19 disease, in a symptomatic than asymptomatic form, was assumed as a control variable. All the relationships between the variables are indicated by a completely standardized β. As represented in Figure 1, positive affect: (1) shows a direct effect on spiritual wellbeing and flourishing; (2) does not have any direct relationship with PTSD symptoms, a negative affect; (3) has a direct effect on PTSD symptoms.

We tested the mediational hypothesis through an estimation of the significance of the indirect effects, using the bootstrapping method. The results, reported in Table 2, show that the direct path from positive affect to PTSD symptoms is not significant (β = −0.003, *p* = 0.94); conversely, the direct path from negative affect to PTSD symptoms is significant (β = 0.34, *p* < 0.001). Therefore, Hypothesis 1a is partially confirmed; moreover, the indirect relationship between positive affect and PTSD symptoms, mediated by the fear of COVID-19, is not significant (β = −0.009, *p* = 0.70). However, the indirect relationship between negative affect and PTSD symptoms, mediated by the fear of COVID-19, is significant (β = 0.30, *p* < 0.001), partially confirming Hypothesis 1b. The path from positive affect to spiritual wellbeing is significant (β = 0.43, *p* < 0.001), unlike the path from negative affect to spiritual wellbeing, which is not significant (β = −0.14, *p* = 0.12), partially confirming Hypothesis 2a. Fear of COVID-19 plays a mediation role in the relationship between negative affect and spiritual well-being (β = 0.19, *p* < 0.001), while the indirect effect of positive affect on spiritual well-being through the mediation of fear of COVID-19 is not significant (β = −0.005, *p* = 0.73). Hypothesis 2b is partially confirmed. Finally, positive affect has a direct relationship with flourishing (β = 0.55, *p* < 0.001), while negative affect is not directly related with flourishing (β = −0.12, *p* = 0.09), partially confirming Hypothesis 3a; moreover, as the path from positive affect to fear of COVID-19 is not significant, the indirect effect is not found (β = −0.004 *p* = 0.71). On the contrary, the indirect effect of negative affect on flourishing through mediation of the fear of COVID-19 is significant (β = 0.13, *p* = 0.01). These results partially confirm Hypothesis 3b.

## 5. Discussion

We aimed to explore whether the fear of COVID-19 was related to both positive and negative affects, and whether this influenced positive (spiritual wellbeing and flourishing) or negative states (PTSD symptoms). We found that the fear of COVID-19 fully mediates the relationship between negative affect and spiritual wellbeing and flourishing; moreover, fear of COVID-19 partially mediates the relationship between negative affect and PTSD symptoms. Regarding the positive affect, the relationships with positive outcomes are only direct, and we did not find any mediation role played by fear of COVID-19.

Regarding the relationship between positive and negative affect and PTSD symptoms, some authors studied the role that positive and negative affects play in PTSD symptoms [98], and negative affect may be a risk factor for the development PTSD symptoms following a disaster [99,100,101]. More recently, Weems and colleagues [102] showed that young people with high negative affect are more at risk of developing PTSD symptoms than those with low levels, but their findings were likely due to shared variance with other dimensions. Similarly, in our research, negative affects were related to PTSD symptoms, both directly and indirectly, via fear of COVID-19. This means that fear could play an important role in the development of PTSD symptoms during the coronavirus pandemic, especially in individuals that predominantly experience negative feelings.

Regarding the relation between affects and spiritual well-being, the literature shows that spiritual wellbeing also positively correlates with positive affective states [83,84] and with relief of suffering [85] during the COVID-19 pandemic. In this regard, interest in the relationship between spirituality and affects has increased in recent years [103,104,105,106,107]. In our study, we found that positive and negative affects play a different predictive role in spiritual well-being: positive affects have a direct effect on spiritual well-being, while negative affects do not directly predict spiritual well-being. Moreover, consistent with the previous literature on fear [8,9], our study shows how the fear of COVID-19 plays a role in the way negative affects impact spiritual wellbeing. In our study, spiritual wellbeing is understood not only in a religious sense, but also as the meaning of life and the quality of relationships with others [72]. This could be explained by supposing that individuals, due to their fear of COVID-19, are more encouraged to reflect on the religiosity and meaning of life, also tending to appreciate relationships with others more than in the past. On the contrary, fear of COVID-19 does not interfere in the relationship between positive affect and spiritual well-being. Positive affect could probably act as a protective factor. 

The relationship between positive and negative affect and flourishing has been extensively studied in the literature [23,73,108], and some authors have pointed out that flourishing is a balance between these two affects [109,110]. However, in our study, positive affect has a direct effect on flourishing; on the contrary, negative affect does not predict flourishing. Thanks to the innovative element that we included in this study, the fear of COVID-19, we have shown that the latter could play an important role between people’s negative affects and their wellbeing, strengthening or reducing the meaningfulness of life through the experience of fear. Contributing to the exposed framework [44,70,74], while the previous literature has shown that positive emotions can be considered as both markers of flourishing [23] and enhancers of flourishing [68], our study reveals that negative emotions can predict eudaimonic wellbeing, or flourishing, through the mediation of fear of COVID-19 during the particular period of quarantine. As well as for spiritual well-being, the fear of COVID-19 can likely encourage people to reflect on the sense and purpose of life, increasing the meaningfulness of life.

The pandemic situation and the consequent restrictive measures issued by governments to contain it may have caused unexpected/rapid and profound/radical changes in people’s lives, eliciting feelings and experiences of fear, uncertainty and anxiety [6,12,111]. It is widely known that fear is a fundamental adaptive defense mechanism for survival. However, if it exceeds certain levels, it becomes harmful and can lead to the development of various psychiatric disorders [112,113]. On the one hand, fear is one of the main factors involved in PTSD symptoms [114,115], even during the COVID-19 pandemic situation [116] and, specifically, fear of COVID-19 can be associated with a wide range of mental health problems, such as anxiety, traumatic stress, and distress [117]. On the other hand, the literature on the relationship between fear of COVID-19 and well-being shows conflicting data: fear of COVID-19 has not been shown to have a direct link with quality of life [118], but in other studies it predicts lower levels of well-being [119].

Our study contributes to enriching the scientific knowledge of the effects that fear of a disease can have on the life of individuals: in fact, fear during the coronavirus pandemic intervenes in the relationship between negative affects on the one hand, and the development of traumatic stress symptoms or individual wellbeing on the other hand.

## 6. Limitations

The limitations of the present study concern the following: first, the choice of a convenience sampling that does not allow for consideration of the study sample as representative of the population; second, the use of self-report tools that can affect social desirability, response set and reaction phenomena to the object, where the interviewee might not react to the statements but to the topics covered; third, the study is cross-sectional, meaning we cannot establish causal relationships between the variables and exclude the risk of reverse causality; fourth, the study was conducted at the beginning of the COVID-19 pandemic. Indeed, the influence of fear of COVID on the dimensions of positive psychology may have varied over time.

## 7. Implications for Future Studies and Practice

Future longitudinal studies could test a more comprehensive model according to what is exposed; indeed, it would be interesting to explore if, at the same levels of negative emotions experienced, fear of COVID-19 can cause lower levels of spiritual wellbeing and flourishing and more PTSD symptoms.

Finally, as the social, economic and individual costs of mental disorders are high, it is important to develop psychological support actions for the management of fear of COVID-19, together with support actions for those who are infected or in quarantine, which are already present in many countries. More specifically, developing psychological support programs aimed at managing fear of COVID-19 could be essential in reducing the impact that negative emotions can have on eliciting PTSD symptoms and decreasing individual well-being. This is especially true for particularly vulnerable people: old people, people with previous illnesses or adolescents in the process of building their identity. Providing psychological relief is an essential component of assistance for populations who are victims of a global emergency such as the coronavirus pandemic.

## 8. Conclusions

The results of the present study can be read in the framework of positive psychology. Indeed, positive affects can be directly related to spiritual wellbeing and flourishing, while negative affects can be indirectly related to spiritual wellbeing, flourishing, and levels of PTSD, through mediation of fear of COVID-19. Therefore, to prevent post-traumatic stress symptoms and achieve greater psychological well-being, in the same way that it is useful to promote the acceptance of all emotional experiences [120], understanding that positive emotions can co-exist with negative [60] is equally useful in supporting people in managing the fear of COVID-19.

Moreover, considering that, as some research shows [45,46], positive affect can also produce positive effects in coping with future stressful events, increasing the experience of this could be even more important in this particular period (still an emergency): in fact, the onset of COVID-19, as well as the continuous fluctuations in the pandemic situation (different peaks in sick people during recent years, but also uncertainty in the information provided by the media, fear of the vaccines, etc.), can represent a source of the resurgence of fear or a further stressful event at each new change.

## Figures and Tables

**Table 1:** Descriptive and correlations between the variables.

		M	SD	1	2	3	4	5	6
1.	Positive affect	31.19	7.57	1					
2.	Negative affect	24.74	8.08	−0.41 ***	1				
3.	Fear of COVID-19	9.00	3.56	−0.28 ***	0.66 ***	1			
4.	PTSD symptoms	36.13	11.65	−0.26 ***	0.64 ***	0.68 ***	1		
5.	Spiritual wellbeing	59.24	10.47	0.41 ***	−0.12 *	0.07	0.01	1	
6.	Flourishing	43.70	7.68	0.54 ***	−0.21 ***	−0.04	−0.07	0.63 ***	1

**Note.** PTSD = Perception of PTSD symptoms; *** *p* < 0.001; * *p* < 0.05.

**Table 2:** Effects of positive and negative affect on PTSD, spiritual wellbeing and flourishing through COVID-19′s fear (completely standardized β).

Paths	Indirect Effect		Direct Effect		Total Effect	
	β	C.I. 95%	β	C.I. 95%	β	C.I. 95%
Positive affect—fear of COVID-19—PTSD symptoms	−0.009	−0.09, −0.06	0.003	−0.15, 0.16	−0.005	−0.16, 0.14
Positive affect—fear of COVID-19—Spiritual wellbeing	−0.005	−0.09, 0.40	0.43	0.42, 0.79	0.43	0.43, 0.76
Positive affect—fear of COVID-19—Flourishing	−0.004	0.05, −0.22	0.55	0.44, 0.68	0.55	0.44, 0.66
Negative affect—fear of COVID-19—PTSD symptoms	0.30	0.31, 0.55	0.34	0.32, 0.67	0.64	0.78, 1.06
Negative affect—fear of COVID-19—Spiritual wellbeing	0.19	0.09, 0.40	−0.14	−0.40, 0.06	0.05	−0.09, 0.22
Negative affect—fear of COVID-19—Flourishing	0.13	0.05, 0.22	−0.12	−0.25, 0.01	0.01	−0.09, 0.11

## Data Availability

The data presented in this study are available on request from the corresponding author.
